# Household Food Insecurity and Demographic Factors, Low Birth Weight and Stunting in Early Childhood: Findings from a Longitudinal Study in South Africa

**DOI:** 10.1007/s10995-022-03555-7

**Published:** 2022-09-28

**Authors:** Abigail Harper, Alan Rothberg, Esnat Chirwa, Winnie Sambu, Sumaya Mall

**Affiliations:** 1 Department of Epidemiology and Biostatistics, University of the Witwatersrand, Johannesburg, 2193 South Africa; 2 School of Therapeutic Sciences, University of the Witwatersrand, Johannesburg, 2193 South Africa; 3 Medical Research Council Gender and Health Research Unit, Pretoria, 0002 South Africa; 4 School of Economics, University of Cape Town, Cape Town, 7701 South Africa

**Keywords:** Periconceptional nutritional status, Low birthweight, Stunting, Food insecurity, Dietary diversity, Food expenditure, LMIC

## Abstract

**Background:**

Low birthweight (LBW) as well as early childhood stunting are risk factors for increased childhood morbidity in low-and middle-income countries (LMIC). The Covid 19 pandemic has exacerbated food insecurity and unemployment globally, prompting concerns for maternal and child health.

**Objectives:**

We used data from the great recession of 2008 to examine the relationship between household food security and other risk factors with LBW and stunting using a longitudinal sample of South African women and their offspring.

**Methods:**

Food security indicators, alcohol use, blood pressure and other characteristics were examined in relation to LBW (≤ 2500 g), stunting (height for age ≤ 2SD) and severe stunting (height for age ≤ 3SD). Regression modelling with clustering at maternal ID level were employed to adjust for maternal characteristics and women who gave birth more than once during the reference period.

**Results:**

Birthweight data were available for 1173 children and height for age 1216 children. The prevalence of LBW was 14.7% while stunting and severe stunting was 17.8% and 14.5%. Child hunger in the household, maternal hypertension and alcohol use were associated with low birthweight. Food expenditure below the Stats SA poverty line and low dietary diversity was associated with stunting and severe stunting respectively. Maternal height and low birthweight were associated with both stunting and severe stunting.

**Conclusions for Practice:**

Interventions that can improve household food security and nutritional status during the periconceptional and antenatal period may reduce the prevalence of low birthweight and subsequent stunting in low- and middle-income countries.

## Significance

*What is Already Known on this Subject?* Household food insecurity is associated with stunting in cross-sectional studies but less is known about the impact of periconceptional and antenatal food insecurity in relation to low birthweight. 

*What this Study adds?* Utilizing longitudinal data, this study found that women who reported a child in the household going hungry were significantly more likely to deliver a low birthweight infant. Low food expenditure and low dietary diversity in the periconceptional period were also associated with stunting and severe stunting among children 5 years later. Interventions to improve periconceptional food security may reduce LBW and subsequent stunting.

## Introduction

Pregnancy and the postpartum period are vulnerable times for women in both high and low and middle income (LMIC) countries. In LMIC many pregnant women live in poverty and may experience a number of stressors including intimate partner violence, economic hardship and food insecurity (Fisher et al., 2012; van Heyningen et al., 2016). The Covid 19 pandemic and the subsequent economic fallout has increased rates of food insecurity and unemployment globally but the long-term implications for maternal and child health are still unfolding. A recent study of pregnancy and birth outcomes in LMIC during the pandemic noted a decrease in antenatal care but no increase in low birthweight (Naqvi et al., 2022). Data from the great recession in 2008 may provide some insights into how social and economic shocks impact maternal and child health. Studies from Portugal and Spain both observed a significant increase in low birthweight during the years of the recession (Kana et al., 2017; Teran et al., 2018).

In the South African context, studies of two longitudinal cohorts have observed associations with LBW in mothers who smoke or drink alcohol as well as those with overweight or obese BMI (Budree et al., 2017; Jeena et al., 2020). An unpublished thesis study among pregnant women in an urban area of South Africa found no association with household food insecurity and low birthweight while another birth cohort study in South Africa found that food insecurity was associated with lower infant gestational age (Zar et al., 2019). In South Africa and other countries undergoing a nutrition transition, the association between food insecurity and birth outcomes is further complicated by high levels of maternal overweight and obesity, a separate risk factor for pregnancy and birth complications (Melchor et al., 2019).

These findings, albeit inconsistent, suggest that preconception and antenatal food insecurity may be related to adverse child health outcomes. A review of the literature suggests that there are few longitudinal data on preconception food insecurity and low birthweight in South Africa. This study aimed to fill the gap by examining the relationship between maternal demographic and antenatal characteristics including household food insecurity indicators during the periconceptional and antenatal period with birthweight and stunting in the first 5 years of life using data from a longitudinal population-based survey.

## Methods

The South African National Income Dynamics Study (SA-NIDS) is a nationally representative government funded panel survey of over 28,000 individuals in 7300 households across South Africa. The study aims to track post-apartheid inequality and poverty over time. NIDS utilized a stratified two-stage cluster sample design to randomly select 400 of Statistics South Africa’s 3000 primary sampling units (PSUs) for inclusion in the surveys (Leibbrandt et al., 2009).

The components of the secondary analysis on which this paper is based are described below:

### Sample

**Fig. 1:**
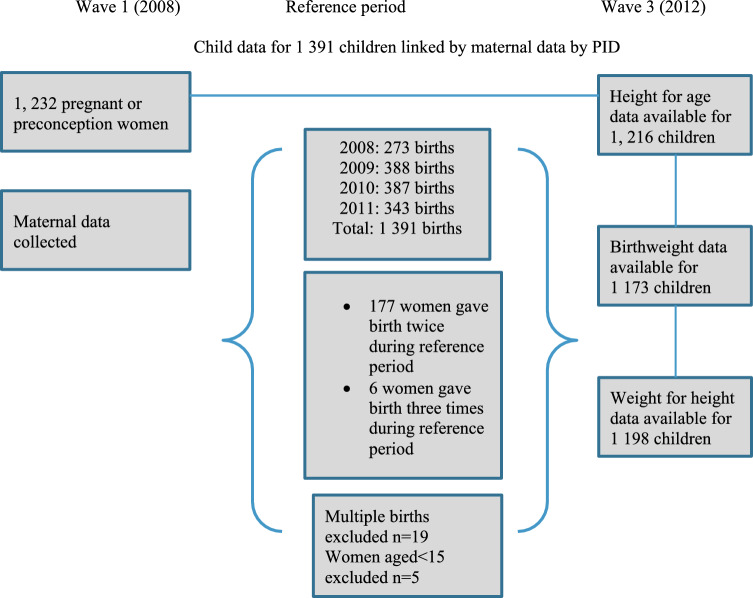
Sample flowchart

The current paper utilized data from Wave 1 and 3 of NIDS. Maternal data were collected in wave 1 (2008) as subsequent waves of NIDS did not collect information on food security. The data from women who became pregnant between Wave 1 and 2 of the study were linked with the data of their children from Wave 3 conducted in 2012. Brief intervals between pregnancies and delivery are a risk factor for low birthweight so we chose to include women who gave birth more than once during the reference period (Bauserman et al., 2020; Blencowe et al., 2019) We linked data of 1208 women with 1391 children born between 2008 and 2011. During data collection, most women (78%) were periconceptional while 21% were in the antenatal stage. The mean time to birth from data collection in 2008 was 21 months (Fig. 1).

### Inclusion and Exclusion Criteria

We limited our analysis to singleton births of women between 15 and 44 because younger adolescents and older mothers are both at increased risk of obstetric complications and LBW (Blencowe et al., 2019). Data on children were limited to those born between 2008 and 2011 to ensure proximity to the time of exposure. We excluded children who had already been born at the time of data collection in Wave 1 (2008) and multiple births. At the time of data collection in Wave 3, children were aged between 4 months and 55 months with a mean age of 30 months.

### Ethical Statement

The SA-NIDS was approved by the Ethics Committee of the Commerce Faculty, University of Cape Town, and the de-identified datasets are publicly available. Ethics approval for this secondary analysis was obtained from the University of the Witwatersrand Research Ethics Committee (protocol number M1909101).

### Measures

#### Food Insecurity

The definition of food security is evolving over time and the past 2 decades have seen a shift from indicators like anthropometry to more subjective measures of food security such as hunger (Coates et al., 2013). Although this study did not include a validated measure of food security, it did examine several household level indicators that literature suggests exist on a spectrum of food security. We will hereinafter refer to these as food security indicators for brevity. The five indicators are: adult and child hunger in the household, household food sufficiency, dietary diversity and monthly per capita food expenditure below the Stats SA food poverty line of R274. In 2021, this inflation adjusted amount was equivalent to R 624 or roughly 43.3 USD.

### Hunger and Nutrition Indicators

#### Adult and Child Hunger

Questions on household hunger were asked separately for adults and children. In the past 12 month did an adult/child in the household go hungry? Responses were never, seldom, sometimes, often or always.

#### Household Food Sufficiency

In the past 12 months please describe the household food consumption in relation to household’s needs. It was less than adequate, it was just adequate, it was more than adequate.

#### Dietary Diversity

A household dietary diversity score (HDDS) based on 30-day recall was calculated using the Food and Agriculture Organization (FAO) guidelines (Kennedy & Ballard, 2010). The HDDS is comprised of 32 individual food types and 12 different food groups. We used a mean split to classify households as having low dietary diversity (score < 9) or high dietary diversity (score > 8). We examined dietary diversity as both a continuous variable and binary variable (Kennedy & Ballard, 2010).

#### Food Expenditure

Per capita food expenditure was calculated by dividing total household monthly food expenditure by the number of household members. Income and expenditure data were imputed by the NIDS team. We used the Statistics South Africa food poverty line cut off of R 274 per capita for 2008 (equivalent to 42.16 USD using Stats SA most recent food poverty line for 2021).

### Covariates for the Birthweight Model

In addition to the primary exposures of food security indicators, we included maternal demographic factors associated with LBW in the literature. These included maternal age, parity, maternal height, BMI categories, years of education, alcohol and tobacco use, depression status, employment and blood pressure (Blencowe et al., 2019). At the household level we included geotype.

#### Alcohol Use

Respondents were asked how often they consumed alcohol. Due to the low number of responses among women who reported drinking alcohol in any quantity, we created a binary indicator by combining women who reported that they never drank or no longer drink into a single category that we classified as not consuming alcohol. Women who reported drinking in any quantity were classified as consuming alcohol, regardless of their pregnancy status.

#### Tobacco Use

Respondents were asked if they smoked cigarettes and we used this binary indicator to classify women as smokers or non-smokers, regardless of their pregnancy status.

#### Depression

Depressive symptoms were measured using the CES-D (Center for Epidemiologic Studies Depression Scale). We used a cut-off of 12 to define depression in this sample (Baron et al., 2017).

#### Employment

Women were categorized as employed or unemployed. Women who were classified as not economically active or seeking employment were also classified as unemployed.

#### Education

Maternal education was examined as a continuous variable with each unit representing 1 year of education. Maternal education in this sample ranged between 9 and 18 years with a mean of 9.86 years.

### Covariates for the Stunting Model

In addition to the food security indicators we examined maternal height, years of education, low birthweight, household size, geotype, child’s age in months, child’s sex and whether child received a child support grant (CSG). This is a government funded monthly disbursement of R 480 ZAR (equivalent to 28.8 USD in 2022) to the primary caregivers of children and is intended to purchase food, school supplies and other essentials.

### Primary Outcomes

The primary outcome is a binary measure of LBW (≤ 2500 g). We included births that were recorded as 2500 g because although birthweight data are normally distributed, heaping birthweight measurement at 2500 g is common practice in the public sector in LMIC to avoid the need for further interventions among LBW infants. In addition, this rounding of birthweight data also occurs due to digit bias for numbers that end in 0 or 5 (Blencowe et al., 2019; Gladstone et al., 2021). The secondary outcome is childhood stunting (height for age ≤ 2SD) and severe stunting (height for age ≤ 3SD) in the first 5 years of life calculated using the WHO child growth standards (WHO, 2008).

### Statistical Analyses

Several steps were undertaken to complete the analyses. These accounted for both individual and household level data. Logistic regression modelling was used to examine the relationship between low birthweight or stunting and maternal risk actors. The standard errors in the logistic regression models were estimated using the clustered robust method to account for the clustering due to multiple births. Initial bivariate analyses were conducted to explore covariates associated with LBW, stunting and severe stunting. All variables in the bivariate analyses were added to the model and the variables that were no longer significant were removed from the final model. Time to birth from the time of data collection was adjusted for in the final birthweight model. We conducted separate analyses among women who gave birth more than once during the reference period to examine if pregnancy spacing was associated with low birthweight but we found no relationship and all women were thus included in the final model.

We used logistic regression for the final LBW model and multivariable regression for the final stunting model in children aged 4–55 months. All analyses were conducted using Stata (version 15).

## Results

### Sample Characteristics

We linked the data from 1208 mothers with the data from their children (N = 1391) who were born between Wave 1 and 2 (2008–2011). Of this sample, 84.5% (1173) children had birthweight data, 87.4% (1216) had height for age data and 86.1% (1198) had weight for height data. Both birthweight and  height for age scores were available for 71.7% (998) children. Maternal characteristics are presented in Table 1.

**Table 1:** Maternal and child socio-demographic characteristics of the sample with birthweight data

Maternal variable (wave 1)	Categories	Overall % (n)
Race* (N = 1016)	Black	82.2 (835)
	Mixed race	15.3 (156)
	Indian and White	1.5 (25)
Age Intervals (N = 1016)	15–19	30 (302)
	20–29	46.2 (470)
	30–39	22.2 ( 225)
	40+	1.7 (17)
Geotype (N = 1016)	Traditional*	42.3 (430)
	Urban	49.7 (505)
	Farm	8 (81)
Employment Status (N = 948)	Unemployed/not economically active	73.5 (697)
	Employed	26.5 (251)
Marital Status (N = 962)	Married/living with partner	24.6 (236)
	Divorced or widowed	1.2 (13)
	Never married	74.1 (713)
Maternal BMI (N = 1016)	Underweight	9.5 (96)
	Normal	43.2 (439)
	Overweight	27.1 (275)
	Obese	20.3 (206)
Ever given birth (N = 965)	Yes	59.5 (574)
	No	40.5 (391)
Depressed (N = 1016)	Yes	16.1 (164)
	No	83.9 (852)
Child Variable (wave 3)		
Gender (N = 1173)	Male	47.3 (618)
	Female	52.7 (618)
Child grant recipient (N = 1213)	Yes	77.3 (907)
	No	22.7 (266)

*We use these categories developed by the South African Apartheid System. Our intention is not to reinforce the differences between races but rather awareness that there are disparities in health reflected by race

Traditional geotype denotes communally-owned land under the jurisdiction of traditional leaders. Settlements within these areas are villages

Most women in the sample were of Black African ethnicity and resided in urban areas. The multiple social and economic vulnerabilities of our sample are visible in the high proportion of adolescents (30%) and unemployment (73.5%) among these women. In addition, most respondents were unmarried (74.1%) and did not live with a partner. Almost half of respondents were overweight or obese (47.4%) and 59.5% had previously given birth.

Table 2 presents the various food security indicators for households with birthweight data. Among these, the most reported food security indicator was per capita food expenditure below the Stats SA poverty with 81.2% of households falling into this category. Mean food expenditure was R215 per capita, considerably below the poverty line. The next most common indicator was insufficient household food adequacy in the past 12 months which was reported in 44% of households. The mean dietary diversity score was 9.1 with a standard deviation of 2.2 and 35.7% of households had low dietary diversity ( a score of less than 9).

**Table 2:** Food security indicators for the sample with birthweight data

Indicator	% (n)
*Child hunger in the past year*	
Never	68.7 (745)
Seldom	8.6 (93)
Sometimes	18.6 (202)
Often	3.6 (39)
Always	0.5 (5)
*Adult hunger in the past year*	% (n)
Never	65.6 (767)
Seldom	8.4 (98)
Sometimes	21.5 (251)
Often	4.1 (48)
Always	0.4 (5)
*Household food adequacy*	% (n)
Less than adequate	44 (513)
It was just adequate	43 (501)
It was more than adequate	13 (152)
*Food expenditure*	% (n)
Below poverty line	81.2 (953)
Above poverty line	18.8 (220)
*Dietary diversity*	Mean, SD
Continuous score 1–12	9.1 (2.2)
	% (n)
Low dietary diversity < 9	35.7 (418)

Table 3 presents the anthropometric characteristics of children measured in Wave 3. Height-for-age scores were available for 1216 children and weight-for-height scores were available for 1198 children. The prevalence of stunting and severe stunting was 17.8% and 14.4% respectively with 216 children classified as stunted (height for age score < −2 SD) and 176 as severely stunted (height for age score < − 3 SD). A total of 18.4% of children classified as overweight or obese (weight for height Z score > 2 SD) while 6% of children were wasted (weight for height Z score < −2 SD). The most severe growth restriction as well as the highest proportion of overweight and obesity occurred in the 4–24-month age range and subsequently declined. Among children in the 4–24-month category, 15.1% were stunted and 24.7% were severely stunted. More than a quarter (26%) of children in this age group were also overweight or obese (Table 3).

**Table 3:** Child anthropometry by age category

Indicator	Child age category			
	4–24 months % (n)	25–48 months % (n)	49–55 months % (n)	Total % (n)
*Height for age*				
Normal height	60.2 (231)	71.6 (537)	68.3 (56)	67.8 (824)
Stunted	15.1 (58)	19.2 (144)	17.1 (14)	17.8 (216)
Severely stunted	24.7 (95)	9.2 (69)	14.6 (12)	14.4 (176)
Total	100 (384)	100 (750)	100 (82)	100 (1216)
*Weight for height*				
Normal weight	66.9 (263)	79.5 (582)	83.6 (61)	75.6 (906)
Wasted or severely wasted	7.1 (28)	5.5 (40)	5.5 (4)	6 (72)
Overweight or obese	26 (102)	15 (110)	10.9 (8)	18.4 (220)
Total	100 (393)	100 (732)	100 (73)	100 (1198)

Birthweight information was available for 1173 children. Mean birthweight was 3102 g with a SD of 531 g. The prevalence of LBW in the sample was 14.7% (a total of 172 babies). In bivariate analyses, overweight BMI, maternal hypertension, child hunger in the household and tobacco use were significantly associated with low birthweight. Variables that remained significant in the final multivariate regression model were households that reported a child ‘sometimes’ going hungry, stage 1 maternal blood pressure, and women who reported drinking alcohol in any quantity (Table 4).

**Table 4:** Unadjusted and adjusted measures of the effect of maternal characteristics on low birthweight logistic regression model

Bivariate regression model	% (n)	Unadjusted OR, Confidence Intervals	p value
Overweight BMI	27.1 (275)	0.52 (0.29–0.90)	0.021
Stage 1 maternal blood pressure	18.1 (252)	1.60 (1.06–2.40)	0.025
Child hunger in household	18.8 (243)	1.59 (1.06–2.38)	0.029
Adult hunger in household	21.5 (251)	1.29 (0.87–1.91)	0.199
Household food less than adequate	44 (513)	1.50 (0.85–2.65)	0.162
Smokes tobacco	7.6 (84)	1.76 (1.03–2.98)	0.042
Drinks alcohol	10.8 (143)	1.57 (0.96–2.56)	0.070
Rural Geotype	42.3 (430)	1.00 (0.71–1.41)	0.995
Depressed mother	16.1 (164)	0.89 (0.56–1.42)	0.628
Employed mother	25.7 (336)	0.95 (0.64–1.39)	0.775
Maternal age	NA	1.02 (0.99–1.04)	0.228
Maternal education in years	NA	0.99 (0.93–1.05)	0.740
Maternal height	NA	0.99 (0.97–1.01)	0.295
Parity	NA	0.98 (0.86–1.10)	0.687

Multivariate regression model	% (n)	Adjusted OR, Confidence Intervals	p Value
Drinks alcohol	10.8 (143)	1.78 (1.08–2.94)	0.023
Stage 1 maternal blood pressure	18.1 (252)	1.61 (1.02–2.51)	0.038
Child hunger in household	18.8 (243)	1.53 (1.01–2.35)	0.049

### Bivariate Analyses

In bivariate analyses, food expenditure below the poverty line doubled the risk of stunting (RRR = 1.97) while low dietary diversity increased the risk of severe stunting (RRR 1.87) (Table 5). Male children were more likely to be severely stunted than female children although there were no gender differences among stunted children. Somewhat surprisingly, child hunger in the household was not associated with stunting. Low birthweight was associated with stunting but not severe stunting. Children who received a child support grant were significantly more likely to be stunted than children who received no grant. This suggests that the children in this sample who do receive a grant are more disadvantaged and experience a generally deficient growth environment (Table 5).

**Table 5:** Unadjusted bivariate analyses of the effect of independent variables on stunting and severe stunting in children aged 4–55 months with relative risk ratios

Stunting	Unadjusted relative risk ratio, Confidence Intervals	p Value
Food poverty line (R274)	1.97 (1.24–3.11)	0.004
Dietary diversity score (continuous)	0.97 (0.91–1.04)	0.362
Low dietary diversity (score < 9)	1.07 (0.77–1.47)	0.647
Child hunger in household	1.09 (0.73–1.62)	0.814
Maternal height in cm	0.94 (0.92–0.96)	0.000
Maternal education	0.97 (0.92–1.02)	0.277
Male child	1.21 (0.90–1.62)	0.217
Receives child grant	1.67 (1.11–2.50)	0.013
Low birthweight	1.85 (1.21–2.84)	0.005
Childs age in months	0.94 (0.92–0.96)	0.000
Maternal education	0.97 (0.92–1.02)	0.277
Household size	1.02 (0.97–1.06)	0.469

Severe stunting	Unadjusted relative risk ratio, Confidence Intervals	p Value
Food poverty line (R274)	1.73 (1.06–2.81)	0.028
Dietary diversity score (continuous)	0.90 (0.84–0.97)	0.003
Low dietary diversity (score < 9)	1.87 (1.30–2.55)	P < 0.000
Child hunger in household	1.19 (0.76–1.85)	0.445
Maternal height in cm	0.96 (0.93–0.99)	0.009
Maternal education	0.93 (0.87–0.98)	0.013
Male child	1.54 (1.11–2.12)	0.009
Receives child grant	1.13 (0.76–1.68)	0.534
Low birthweight	1.52 (0.94–2.44)	0.085
Childs age in months	0.97 (0.96–0.98)	0.000
Maternal education	0.93 (0.87–0.98)	0.013
Household size	1.00 (0.96–1.06)	0.862

### Multivariate Regression Model

Variables associated with stunting and severe stunting were combined in a final multivariable regression model (Table 6). In the final model, maternal height and low birthweight were the only variables associated with both stunting and severe stunting with increased maternal height offering a protective effect. The food security indicators that remained significant in the final model were household food expenditure below the poverty line which increased the risk of stunting and low household dietary diversity which increased the risk of severe stunting. Boys were more likely to be severely stunted than girls and the risk of severe stunting decreased with each month of children’s age.

**Table 6:** Multivariate models of adjusted measures of the effect of independent variables on stunting and severe stunting in children aged 4–55 months with relative risk ratios

	Adjusted relative risk ratio, Confidence Intervals multinomial regression model	p value
*Stunting*		
Low dietary diversity	0.74 (0.50–1.11)	0.145
Food poverty line (R274)	2.31 (1.27–4.22)	0.006
Low birthweight	1.71 (1.05–2.78)	0.030
Maternal height	0.95 (0.92–0.97)	p < 0.000
Childs age in months	1.00 (0.99–1.02)	0.762
Male child	1.17 (0.81–1.68)	0.398
*Severe stunting*		
Low dietary diversity	1.57 (1.05–2.34)	0.027
Food poverty line (R274)	1.21 (0.69–2.12)	0.498
Low birthweight	1.76 (1.06–2.93)	0.028
Maternal height	0.96 (0.94–0.99)	0.008
Childs age in months	0.97 (0.96–0.99)	0.002
Male child	1.57 (1.08–2.29)	0.018

## Discussion

This study has revealed important findings about household food insecurity during the preconception and antenatal periods and its association with child health outcomes in the first 5 years of life. We found that women who reported a child in the household sometimes going hungry were significantly more likely to give birth to a low birthweight infant during the reference period. Furthermore, LBW was a significant risk factor for stunting as children aged. These findings highlight the importance of adequate maternal nutrition during the first 1000 days to mitigate the prevalence of low birthweight and subsequent stunting in LMIC.

The prevalence of LBW in the sample was 14.7% and is closely aligned with recent cohort studies of birthweight in South Africa (Budree et al., 2017; Jeena et al., 2020). We found that child hunger in the household was significantly associated with low birthweight but adult hunger was not. Food insecurity is a managed process and longitudinal data suggests that adults will often forego meals themselves to shield their children from hunger (Radimer et al., 1992). Thus, women that reported children going hungry may represent the most disadvantaged and nutritionally deprived households. We also found that the prevalence of low birthweight in this sample was highest during the recession in 2008 (16.4%) and subsequently declined each year to 12.9% by 2011. These differences were not statistically significant, but this may be due to the relatively low sample size. Given that an increase in low birthweight was observed in high income countries it seems plausible that South Africa would also have experienced an increase in LBW as a result of the recession (Kana et al., 2017; Teran et al., 2018).

Although maternal alcohol use was not significantly associated with LBW in bivariate analysis (p = 0.07), once it was added to the final model and we adjusted for the time lag between data collection and birth, women who reported drinking alcohol in any quantity were significantly more likely to deliver a low birthweight infant. In this study, 10.8% of women reported drinking alcohol, a result that is comparable to a cohort study in the Western Cape that observed antenatal alcohol use among 18% of women (Budree et al., 2017). Although our study included both periconceptional and pregnant women, South Africa has one of the highest rates of Fetal Alcohol Syndrome (FAS) globally which suggests that alcohol use continues into the antenatal period for some women. Food insecurity may also increase the risk of drinking among women who face multiple social and economic adversities. A study among urban mothers in South Africa found that food insecurity was strongly associated with postnatal depression as well as hazardous drinking. These findings reinforce the importance of food security for psychological well-being as well being a potential risk factor for hazardous drinking, thereby compounding the risk of low birthweight (Dewing et al., 2013).

Our final model also found that maternal hypertension was significantly associated with LBW, an association that has been observed in a systematic review (Getaneh et al., 2020). Hypertension has been linked to food security, obesity and increased BMI as well as poor quality diets, higher salt intake and chronic stress (Cois & Ehrlich, 2014). Thus, hypertension may lie on the causal pathway between food insecurity and low birthweight.

Our study found a similar stunting prevalence to other South African cohorts that examined linear growth in children age 24–60 months and noted prevalence estimates between 26% and 34% (Budree et al., 2017; Casale et al., 2018). The link between LBW and stunting has been well described in previous literature with an analysis of cohort data from LMIC estimating 2.5–3.5 higher odds of stunting among LBW infants (Christian et al., 2013). In our final multivariable stunting model we found that LBW increased the risk of stunting (RRR 1.71) and severe stunting (RRR 1.76). These findings suggest that some aspects of childhood undernutrition and subsequent linear growth have origins in the foetal period and highlight the importance of maternal nutrition both preconceptionally and antenatally (Black et al., 2008; Blencowe et al., 2019; Christian et al., 2013). Increased maternal height also offered a protective effect against stunting and severe stunting in the final model. This is well documented as stunting is a recurrent process and mothers who were themselves stunted are more likely to have stunted offspring (de Onis & Branca, 2016).

Food expenditure below the poverty line was associated with stunting in the final mode. Such data are a useful proxy for food security although these results are less precise than some other measures of food insecurity (Coates et al., 2013). In the final model, low dietary diversity was associated with severe stunting. This has emerged as a risk factor for stunting in studies from LMIC is often associated with child anthropometry across geographical regions (Headey & Ecker, 2013). Somewhat surprisingly, child and adult hunger were not associated with stunting in this sample. This is of as interest as most large surveys currently employ access and hunger-based measures to measure food security at the national scale.

Although food security is not static and subject to change, the use of longitudinal data is one of the strengths of this study and these findings indicate that chronic household food insecurity during the periconceptional and antenatal period likely extends into the early years of childhood and is associated with both low birthweight and restricted linear growth. These findings are reinforced by a study in Bangladesh with a 5 year recall period which found that household food security tended to be relatively stable and preconception food insecurity continued into the antenatal period (Chowdhury et al., 2018). Maternal malnutrition (both underweight and obesity) is associated with adverse child growth outcomes including low birthweight, macrosomia, fetal growth as well as child HAZ at 2 years of age (Melchor et al., 2019; Young et al., 2018).

We also found that the prevalence of severe stunting decreased as children grew older, this is not surprising as previous studies suggest that stunting tends to peak around 24 months and subsequently decrease (Prendergast & Humphrey, 2014). In addition, boys were more likely to be severely stunted than girls. This finding has been observed in a a systematic review of sex related differences in undernutrition (Thurstans et al., 2020). The reasons for this may be both social as well as biological. Girls may spend more time at home and have more regular access to food than boys and boys may be weaned earlier than girls (Thurstans et al., 2020). Biologically, boy infants are also more susceptible to infections and birth complications in early childhood which can predispose them to stunting as they age (Sawyer, 2012).

The emphasis on child growth and development in the first thousand days is based not only on the magnitude of growth restriction that occurs in this time period but on the adverse consequences throughout the life course including reduced cognitive function and an increased risk of chronic disease in adulthood (de Onis & Branca, 2016). In this sample, 25% of children aged 24 months and under were severely stunted and many will never reach their full growth potential as a result (Casale et al., 2020). Thus, interventions that improve periconceptional maternal nutritional status are particularly important in settings where stunting is prevalent. A multi country preconceptional maternal nutritional trial found that the provision of nutritional supplements pre conception and in the early stages of gestation significantly improved infant growth outcomes in the first six months of life (Krebs et al., 2020).

### Limitations

Data on gestational age were not available and preterm birth (a leading cause of LBW) could not be included in our analyses (Blencowe et al., 2019; Christian et al., 2013). In the absence of a validated measure for food insecurity this study examined the relationship between various food security indicators and child health outcomes. These indicators were collected at the household level and the questionnaire was answered by oldest woman in the household. This is standard for household level surveys and given that data was not collected at the individual level we don’t feel that this would have introduced substantial bias.

## Conclusion

Reducing the high prevalence of low birthweight and stunting in South Africa and other LMIC is an enormous challenge given the multiple social and biological determinants of health inequity. However, interventions that can improve household food insecurity and maternal nutritional status in the periconceptional period may reduce the prevalence of low birthweight and subsequent stunting (Krebs et al., 2020; Lassi et al., 2020). Improving maternal nutritional status in the first 1000 days is an important first step to mitigate the intergenerational transfer of poor health and developmental outcomes.

## Data Availability

Deidentified data from the South African National Income Dynamics Study (NIDS) can be accessed via the NIDS website (http://www.nids.uct.ac.za/). Alternatively, data that contains confidential information can be accessed via an application process with Datafirst at the University of Cape (www.datafirst.uct.ac.za).

## Code Availability

NA.
